# Phytochemical Combination Is More Effective than Individual Components in Reducing Stress Signaling in Rat Hippocampal Neurons and Microglia In Vitro

**DOI:** 10.3390/ijms232012651

**Published:** 2022-10-21

**Authors:** Derek R. Fisher, Tong Zheng, Donna F. Bielinski, Megan E. Kelly, Danielle S. Cahoon, Barbara Shukitt-Hale

**Affiliations:** United States Department of Agriculture, Agricultural Research Service, Human Nutrition Research Center on Aging at Tufts University, Boston, MA 02111, USA

**Keywords:** EGCG, broccoli sprouts, curcumin, sulforaphane, anti-inflammatory, calcium signaling, microglia, neurons

## Abstract

Age-related decrements in the central nervous system (CNS) are thought to result from: (1) increased susceptibility to and accumulating effects of free radicals and inflammation; and (2) dysregulation in Ca^2+^ homeostasis, which affects numerous signaling pathways. Certain bioactive phytochemicals exhibit potent anti-inflammatory activities which may mitigate these age-related CNS decrements. This study investigated the individual and combination effects of green tea catechin (epigallocatechin gallate, EGCG), curcumin from turmeric, and broccoli sprouts which contain the isothiocyanate sulforaphane on inflammation and dysregulation in Ca^2+^ homeostasis to determine if the individual compounds were working synergistically and/or through independent mechanisms. Rat hippocampal neurons or highly aggressive proliferating immortalized (HAPI) microglial cells were pre-treated for a week with either the individual components or all in combination before inducing Ca^2+^ buffering deficits with dopamine (DA, 0.1 µM for 2 h) or inflammation using lipopolysaccharide (LPS, 100 ng/mL for 18 h), respectively. The EGCG (3 µM) and combination protected against DA-induced deficits in Ca^2+^ buffering (both % of cells that recovered and recovery time, *p* < 0.05). Additionally, the EGCG and combination reduced stress-mediated inflammation in HAPI rat microglial cells by attenuating LPS-induced nitrite release, inducible nitrous oxide synthase (iNOS) expression, and tumor necrosis factor-alpha (TNF-α) release (*p* < 0.05), but not cyclooxygenase-2 (COX-2) expression. Overall, broccoli sprouts (2 µM) and curcumin (1 µM) were not as effective as the EGCG or combination. Further research is needed to determine if dietary intervention with a variety of foods containing compounds such as those found in green tea, turmeric, or broccoli sprouts can play a role in reducing age-related CNS inflammation, microglial activation, and downstream signaling pathways that can lead to neuronal dysfunction.

## 1. Introduction

The central nervous system (CNS) is highly susceptible to age-related increases in free radicals (reactive oxygen and nitrogen species) and inflammation, as well as the dysregulation of Ca^2+^ homeostasis. These increases in vulnerability are thought to contribute to the decline of motor abilities and cognitive performance seen with age [1]. The long-term effects of these age-related decrements from inflammatory insults coincide with the decrease of the body’s natural defense and repair mechanisms through the lifespan, while the dysregulation of Ca^2+^ homeostasis affects numerous signaling pathways [2,3]. Certain foods and their individual constituents have been shown to have protective effects against age-related cognitive and neurochemical/neuronal dysfunction [4]. These neuroprotective effects may be due to the phytochemicals and other components contained in these foods, as they exhibit potent antioxidant, anti-inflammatory, and anti-atherogenic activities that can reduce the age-related sensitivity to free radical damage and/or inflammation [1,5].

Phytochemicals may provide neuroprotection by preventing or delaying chronic inflammation and free radical damage. For example, epigallocatechin gallate (EGCG) in green tea can protect against dopamine (DA)-induced neuronal damage and Aβ-induced cognitive impairment [6,7]. Curcumin, a bioactive phytochemical in turmeric, protects against stressor-induced oxidative stress (OS) in neurons, and upregulates antioxidant and anti-inflammatory defense mechanisms in the brain tissue of animal models [8]. Sulforaphane, an isothiocyanate in broccoli sprouts, protects against neuronal OS, cognitive deficits, and is a potent activator of antioxidant transcription factor Nrf2 [7]. Additionally, these individual phytochemicals have exhibited high antioxidant, anti-inflammatory, autophagy-inducing, and Nrf-2 activating properties in human neurodegenerative diseases, including Parkinson’s and Alzheimer’s disease models [9,10,11,12]. It is possible that combining these individual bioactive plant components could potentially affect and target complementary networks [6]. It is also likely that these different compounds have diverse mechanisms of action, and/or work synergistically, and therefore work more effectively in combination than alone [13].

Because whole foods contain an abundance of phytochemicals, and individual bioactive phytochemicals may work through multiple mechanisms to achieve these preventative effects, it may be important to investigate the effects of combined compounds, rather than singly administered ones [14]. It is possible that individual compounds in the different components may act additively, synergistically, or exert their effects through different and/or independent mechanisms [5,13].

Therefore, in this study, we investigated whether these purported neuroprotective phytochemicals [EGCG, curcumin (CURC), and sulforaphane-containing broccoli sprouts (BSP)] administered alone and in combination (ECB) could enhance calcium buffering in neurons and/or reduce stress signaling (i.e., inflammation) in microglial cells. Hippocampal neurons were examined for their ability to recover following a cellular stressor (DA) application, while microglia were assessed for the extracellular release of nitric oxide (NO) and tumor necrosis factor-alpha (TNF-α), as well as intracellular levels of inducible nitrous oxide synthase (iNOS) and cyclooxygenase-2 (COX-2), following inflammatory stress induced by lipopolysaccharide (LPS). Dopamine was used as an oxidative stressor because DA rapidly oxidizes to form reactive oxygen species and quinones [15]. We have previously shown that cells exposed to DA show reductions in the ability to extrude or sequester Ca^2+^ following KCl stimulation, leading to the dysregulation of Ca^2+^ homeostasis [16].

## 2. Results

### 2.1. Calcium Recovery in Neurons

**Figure 1:**
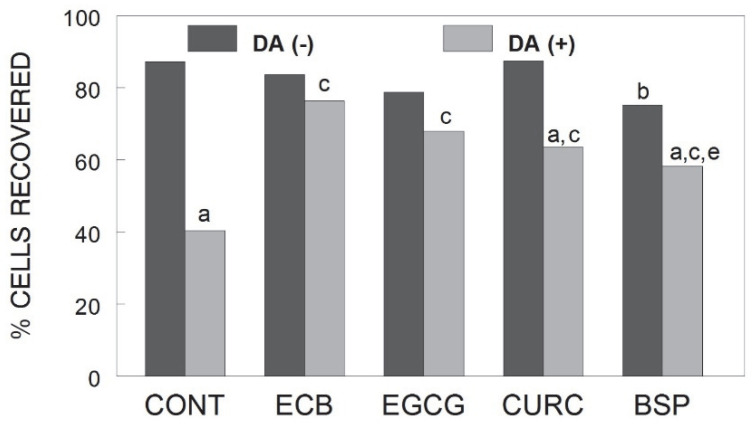
Calcium recovery in E18 rat hippocampal neurons pre-treated with ECB or its component phytochemicals (EGCG, CURC, BSP) and stimulated for 2 h with DA (0.0 or 0.1 mM). Data are expressed as the percentage of cells that return to baseline calcium levels. a = *p* < 0.05 vs. non-DA-treated cells matched for each treatment; b = *p* < 0.05 vs. non-DA-treated control cells; c = *p* < 0.05 vs. DA-treated control cells; e = *p* < 0.05 vs. DA-treated ECB cells; Mann–Whitney U post-hoc. DA = dopamine; CONT = control; ECB = combination compound; EGCG = epigallocatechin gallate; CURC = curcumin; BSP = broccoli sprouts.

Figure 1 shows that the administration of DA significantly reduced the percent of calcium recovery in neuronal cells, and all treatments in DA-treated cells showed increased percent recovery when compared to DA-treated control cells [χ^2^_(11)_ = 96.07 *p* < 0.001]. However, only the ECB combination and EGCG were able to bring the percent recovery back to control levels, as DA-treated cells with PBC and EGCG were not significantly different from their respective non-DA-treated groups (*p* > 0.05). CURC and BSP showed reduced recovery compared to their own non-DA-treated cells (*p* < 0.05). Additionally, BSP showed reduced recovery in non-DA-treated cells compared to non-DA-treated control cells (*p* < 0.05), and when compared to ECB, BSP also showed reduced recovery in DA-treated cells (*p* < 0.05).

**Figure 2:**
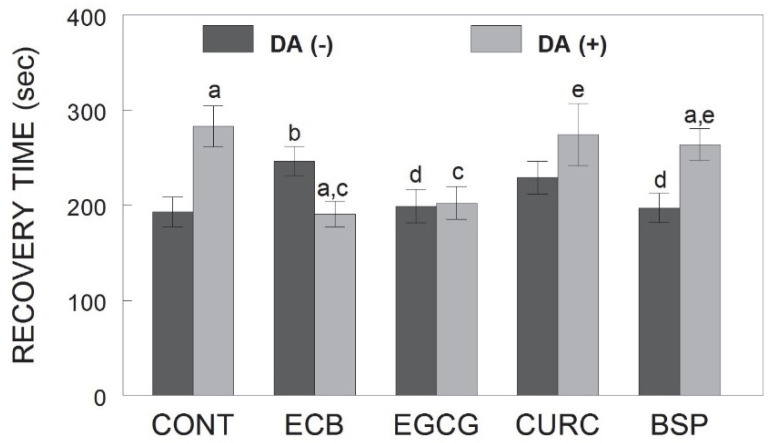
Calcium recovery time in E18 rat hippocampal neurons pre-treated with ECB or its component phytochemicals (EGCG, CURC, BSP) and stimulated for 2 h with DA (0.0 or 0.1 mM). Data are expressed as mean time (seconds) to return to baseline calcium levels after depolarization ± SEM. a = *p* < 0.05 vs. non-DA-treated cells matched for each treatment; b = *p* < 0.05 vs. non-DA-treated control cells; c = *p* < 0.05 vs. DA-treated control cells; d = *p* < 0.05 vs. non-DA-treated ECB cells; e = *p* < 0.05 vs. DA-treated ECB cells; Fisher’s LSD post-hoc test. DA = dopamine; CONT = control; ECB = combination compound; EGCG = epigallocatechin gallate; CURC = curcumin; BSP = broccoli sprouts.

The time to recover from depolarization to 70% of baseline, in those cells that recovered, is shown in Figure 2. The ANOVA showed a significant treatment × DA interaction [F(5, 675) = 5.05, *p* < 0.001]. Although the ECB combination increased recovery time (i.e., slowed recovery) in non-DA-treated cells, compared to control cells (*p* < 0.05), the combination and EGCG in DA-treated cells were able to reduce recovery time compared to DA-treated control cells (*p* < 0.05). Furthermore, in non-DA-treated cells, EGCG and BSP had quicker recovery times compared to the combination, while in DA-treated cells, CURC and BSP had slower recovery times compared to the combination (*p* < 0.05).

### 2.2. Inflammatory Stress Signaling in Microglial Cells

**Figure 3:**
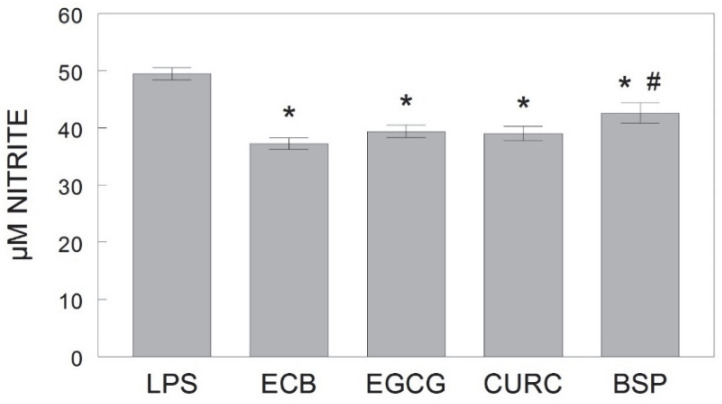
Production of extracellular NO, as measured by μM nitrite, in HAPI rat microglial cells pre-treated with ECB or its component phytochemicals (EGCG, CURC, BSP) and stimulated 18 h overnight with LPS (100 ng/mL). Data are represented as mean ± SEM (μM nitrite) and quantified using Greiss reagent. Each phytochemical pre-treatment was compared against LPS alone and individual components were compared to ECB. Comparative post-hoc analyses were made by Fisher’s LSD post-hoc test with significance at (*) *p* < 0.05 versus LPS or (#) *p* < 0.05 versus ECB. LPS = lipopolysaccharide only; ECB = combination compound; EGCG = epigallocatechin gallate; CURC = curcumin; BSP = broccoli sprouts.

As shown in Figure 3, the individual phytochemical treatments of EGCG, CURC, and BSP, as well as their combination (ECB) significantly attenuated LPS-induced nitric oxide (NO) production in HAPI rat microglial cells [F(6, 49) = 15.01, *p* < 0.001]. NO is a free radical released from microglial cells following activation by inflammatory stressors such as LPS. Although BSP significantly reduced LPS-induced NO production relative to cells treated with LPS only, it was not as effective as the ECB combination, as the NO release was significantly higher with BSP compared to the release following the combination pre-treatment (*p* < 0.05).

**Figure 4:**
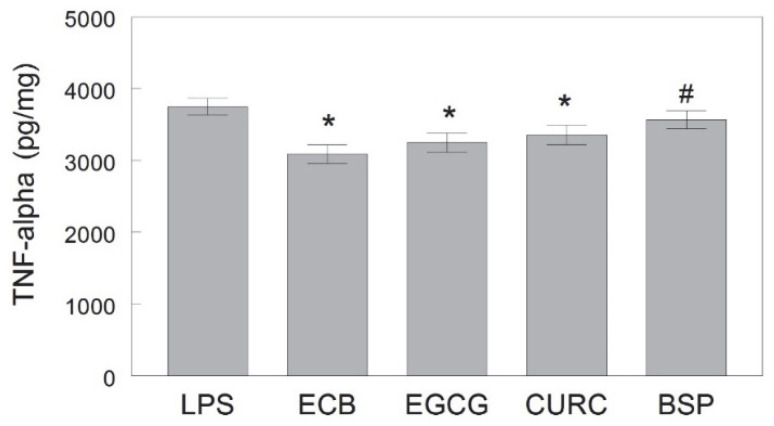
Suppression of TNF-α release in HAPI rat microglial cells pre-treated with ECB or its component phytochemicals (EGCG, CURC, BSP) and stimulated 18 h overnight with LPS (100 ng/mL). Data are expressed as mean ± SEM (pg/mg) as assayed by ELISA. Each pre-treatment was compared against LPS alone and individual components were compared to ECB. Comparative post-hoc analyses were made by Fisher’s LSD post-hoc test with significance at (*) *p* < 0.05 versus LPS or (#) *p* < 0.05 versus ECB. LPS = lipopolysaccharide only; ECB = combination compound; EGCG = epigallocatechin gallate; CURC = curcumin; BSP = broccoli sprouts.

Figure 4 shows that the ECB combination and two of its components (EGCG and CURC) significantly reduced the LPS-induced release of inflammatory cytokine TNF-α in HAPI rat microglial cells [F(6, 105) = 4.78, *p* < 0.001]. Conversely, BSP did not significantly reduce LPS-induced TNF-α levels compared to cells treated with LPS only (*p* > 0.05), and BSP had significantly higher levels of TNF-α than the combination (*p* < 0.05), whereas EGCG and CURC were not different than ECB.

**Figure 5:**
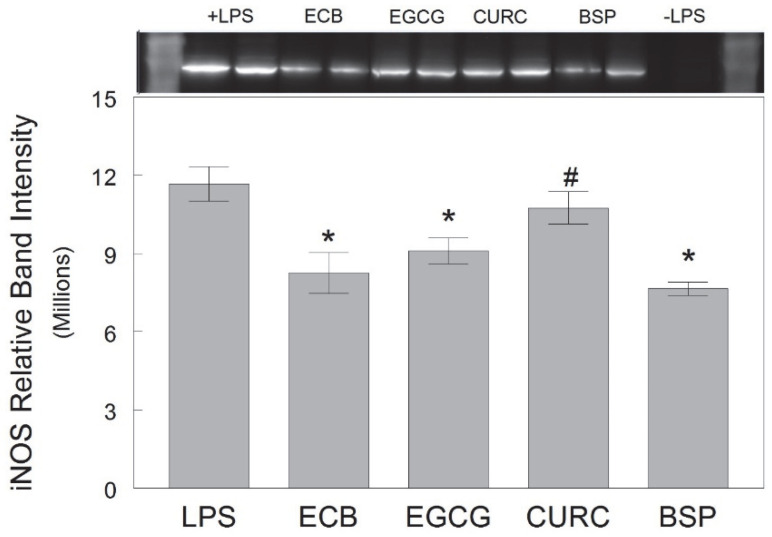
Expression of iNOS in HAPI rat microglial cells pre-treated with ECB or its component phytochemicals (EGCG, CURC, BSP) and stimulated 18 h overnight with LPS (100 ng/mL). Data are expressed as mean ± SEM of the immunoreactive band density per 20 µg total protein as measured by Western blot (top). Each pre-treatment was compared against LPS alone and individual components compared to ECB. Comparative post-hoc analyses were made by Fisher’s LSD post-hoc test with significance at (*) *p* < 0.05 versus LPS or (#) *p* < 0.05 versus ECB. LPS = lipopolysaccharide only; ECB = combination compound; EGCG = epigallocatechin gallate; CURC = curcumin; BSP = broccoli sprouts; −LPS = no LPS.

Our data also showed that the ECB combination and two of its components (EGCG and BSP) significantly reduced the LPS-induced expression of iNOS, a stress-induced enzyme that produces the inflammatory mediator NO, in HAPI rat microglial cells [F(6, 49) = 5.62, *p* < 0.001] (Figure 5). However, CURC did not significantly reduce the LPS-induced iNOS levels compared to cells treated with LPS only (*p* > 0.05), and iNOS was higher in CURC-treated cells compared to ECB-treated cells (*p* < 0.05).

**Figure 6:**
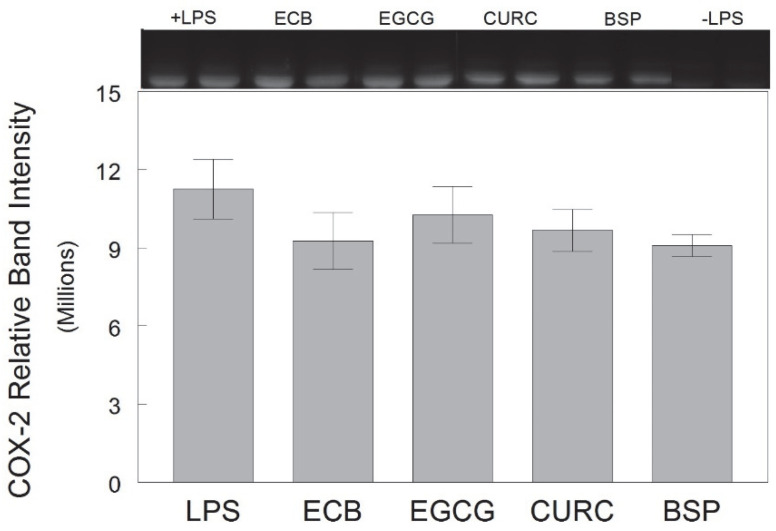
Expression of COX-2 in HAPI rat microglial cells pre-treated with ECB or its component phytochemicals (EGCG, CURC, BSP) and stimulated 18 h overnight with LPS (100 ng/mL). Data are expressed as mean ± SEM of the immunoreactive band density per 20 µg total protein as measured by Western blot (top). Each pre-treatment was compared against LPS alone and individual components compared to ECB. LPS = lipopolysaccharide only; ECB = combination compound; EGCG = epigallocatechin gallate; CURC = curcumin; BSP = broccoli sprouts; −LPS = no LPS.

None of the phytochemical treatments significantly reduced the LPS-induced expression of COX-2 in HAPI rat microglial cells [F(6, 49) = 0.777, *p* = 0.592] (Figure 6). COX-2 is responsible for the formation of prostanoids, which are inflammatory mediators.

## 3. Discussion

The goal of the present study was to measure the effects of the phytochemicals, EGCG, curcumin, and sulforaphane-containing broccoli sprouts, and the combination of them on stressor-induced Ca^2+^ buffering deficits and induced inflammation in rat neurons and HAPI rat microglia cells. Overall, the results showed that the combination of the three phytochemicals and one of their components, EGCG, were consistently effective in protecting against stress-mediated deficits in Ca^2+^ buffering and inflammatory signaling. The other two compounds, curcumin and sulforaphane-containing broccoli sprouts, had partial protective effects, depending on the endpoint measured. The effects of the combination were either greater than or no different from the effects of the individual components. Therefore, the individual components could be acting synergistically to enhance the effects of individual compounds, exerting their effects through different and/or independent mechanisms, or working together on the same pathways. Whether the beneficial effects of the combination of all three phytochemicals are due mainly to EGCG, or if the other two compounds also play an important role, should be examined in future studies.

Dopamine exposure reduced the ability of the hippocampal neurons to extrude or sequester Ca^2+^ following KCl stimulation. Compared to the control, all treatments protected against DA-induced decreases in percent recovery, while only the combination and EGCG were able to return percent recovery to the control levels. Compared to the combined compound, broccoli sprouts and curcumin were not as protective against deficits in Ca^2+^ buffering. The results seen with regards to the greater efficacy of combined phytochemicals were similar to those seen with other multiple polyphenolic-rich treatments, such as blueberries [16,17], where the whole blueberry showed greater protection than its fractions [18]. These results are important because it is thought that at least part of the loss of cognitive function in aging may be dependent upon a dysregulation in calcium homeostasis [18].

Additionally, results indicated that the individual phytochemicals and the combination of the three all showed protective effects against LPS-induced inflammation in microglia; however, the mechanisms and targets may differ for each component. In this study, EGCG was similar in efficacy to the combination in reducing LPS-induced NO, TNF-α, and iNOS, perhaps due to its multiple neuroprotective and neurorescue abilities, including free radical scavenging and anti-inflammatory properties via activation and inhibition of protein kinase signaling pathways [19]. Conversely, although curcumin produced similar beneficial effects as the combination on LPS-induced NO and TNF-α, curcumin alone was not effective in reducing iNOS expression in microglia compared to the combination or LPS-only control, nor was it effective in improving Ca^2+^ recovery time in hippocampal neurons following KCl stimulation. One possible explanation for these results might be the poor bioavailability of curcumin due to its poor absorption, rapid metabolism, and rapid elimination [11].

In the current study, sulforaphane-containing broccoli sprouts significantly attenuated LPS-induced NO production relative to the control, indicating protection against microglia inflammation, but was less effective than the combination at reducing NO. Additionally, broccoli sprouts did not significantly reduce LPS-induced TNF-α release relative to the control as the other components did. However, broccoli sprouts did significantly reduce LPS-induced iNOS compared to the control, and with similar efficacy as the combination, and there was a trend for BSP to reduce COX-2 expression (*p* = 0.084, compared to the LPS-only control). In this study, the protein expression of these two enzymes, i.e., iNOS and COX-2, was analyzed in cell lysates, which may indicate that sulforaphane-containing broccoli sprouts interact with the intracellular inflammatory cascades that lead to the expression of enzymes more than soluble factors in the supernatant (e.g., NO and TNF-α). Sulforaphane is known to increase the expression of the transcription factor Nrf2 (nuclear erythroid 2-related factor 2) and HO-1 (Heme oxygenase-1): an important phase II antioxidant enzyme, and an Nrf2-regulated gene that plays a critical role in the prevention of vascular inflammation [7]. Sulforaphane, when combined with nobiletin, a flavonoid found in citrus fruits, synergistically decreased iNOS and COX-2 protein expression levels and induced heme oxygenase-1 (HO-1) protein expression in LPS-stimulated macrophages [20]. The combination also produced the synergistic (not additive) inhibition on NO production in the cells, and these inhibitory effects were stronger than those produced by either compound alone at much higher doses. However, neither compound alone, nor their combination, inhibited the TNF-α release nor decreased mRNA levels of COX-2, suggesting that they may decrease the protein level of COX-2 by other mechanisms such as modulating translation and/or degradation of protein in LPS-stimulated macrophages [20]. These results support and may help to explain the finding in the present study that broccoli sprouts alone were not as effective at reducing soluble inflammatory mediators (NO, TNF-α) as the combination.

Different mechanisms of action by different phytochemicals may explain how the combination of these phytochemicals could have a greater effect than the average of the effects of each phytochemical separately. The proposed mechanisms by which combining phytochemicals might exert synergistic anti-inflammatory effects include enhanced bioavailability/uptake of each other, increased antioxidant capacity, interactions with gut microbiome, and targeting the same and different signaling pathways [13]. As in the present study, several previous studies have found that combinations of the individual phytochemicals are more effective than these individual compounds alone; however, no study examined the three compounds together. For example, combining broccoli sprouts and green tea polyphenols (e.g., EGCG) led to the synergistic prevention of estrogen receptor-negative mammary tumors through cell cycle arrest and apoptosis induction [14]. A combination of curcumin and EGCG synergistically inhibited the inflammatory mediator NF-κB (nuclear factor kappa-light-chain-enhancer of activated B cells) compared to the individual chemicals in models using prostate and breast cancer cells [21]. Another study showed that the combination of curcumin with sulforaphane had a synergistic effect in inhibiting LPS-induced inflammation in RAW 264.7 macrophages [22]. The authors proposed that the two compounds were working through two distinct pathways, as sulforaphane was most effective in inhibiting iNOS protein expression and curcumin most effective in inducing phase II genes such as HO-1, leading to greater synergism.

This study was conducted in vitro, using cells derived from rats, and therefore further research is needed on how consuming the combination of the three compounds and their components may lead to changes in Ca^2+^ homeostasis and anti-inflammatory effects in vivo. In addition, the doses of the three individual components were not equimolar but instead were selected based on previous unpublished data. Future studies using equimolar doses could clarify if the superiority of EGCG in calcium buffering and anti-inflammatory effects were due to dose differences or underlying mechanisms.

In conclusion, these results support the hypothesis that the combination of three phytochemicals would have a greater beneficial effect on stress-induced calcium buffering and inflammation than their individual components. These findings mirror those found with other compounds of phytochemicals [13] and together show that phytochemicals have the strongest anti-inflammatory effect when combined. These results also align with work on signaling pathways and other markers of inflammation, which are impacted by various phytochemicals [13]. Future research into dietary intervention with compounds such as those found in green tea, turmeric, or broccoli sprouts are necessary to determine if these phytochemicals can play a role in reducing the age-related CNS inflammation, microglial activation, dysregulation in Ca^2+^ homeostasis, and stimulation of immune pathways that reduce neurogenesis and impair cognitive function.

## 4. Materials and Methods

### 4.1. Phytochemical Compounds and Cell Treatments

Individual phytochemical compounds used for treatments were EGCG (Teavigo, Taiyo International, Minneapolis, MN, USA; CAS No. 989-51-5, Chemical Formula C_22_H_18_O_11_), curcumin (CURC, Longvida, Verdure Sciences, Noblesville, IN, USA; CAS No. 458-37-7, Chemical Formula C_21_H_20_O_6_), and broccoli sprouts containing sulforaphane (BSP, BroccoPhane, Bioriginal, Anaheim, CA, USA; CAS No.4478-93-7, Chemical Formula C_6_H_11_NOS_2_). They were prepared by solubilizing the freeze-dried compounds into a stock solution of PBS containing 10% DMSO. Serum-free Dulbecco’s modified Eagle’s medium (DMEM, Invitrogen, Grand Island, NY, USA) was then used to dilute the concentration of DMSO to 0.01%. The resulting solutions were then sterile filtered, aliquoted, and frozen at 20 °C until use. The combination (ECB) was made by adding equal volumes of each phytochemical corresponding to the concentrations used for the individual component: EGCG (3 µM), CURC (1 µM), or BSP (2 µM). Final concentrations and ratios of these components were determined based on the viability of the cells in our pilot experiments, and on data from our collaborators that indicated the neuroprotective effects of these components on mouse neuronal cells. 

### 4.2. Hippocampal Cell Culture

The E18 primary rat hippocampal neurons purchased from BrainBits (Springfield, IL, USA) were plated on Poly-D lysine-coated 8-well chamber slides at a seeding density of 20,000 cells/well in neurobasal media according to the procedures provided (BrainBits Complete Culturing Kit), and maintained as previously described by Joseph and colleagues [16]. The cells were allowed to differentiate for 4 to 5 days at 37 °C with 5% CO_2_ before experimentation. For the experiments, cells were treated with media containing the EBC combination, EGCG, CURC, or BSP alone, or no supplement for 5 days. The media was changed after 3 days and fresh media with or without supplement was added. Following treatments, cells were exposed to 0 or 0.1 mM of OS-inducing DA for 2 h to induce deficits in Ca^2+^ buffering.

### 4.3. Ca^2+^ Imaging

Calcium imaging was conducted as previously described by Joseph and colleagues [16]. Briefly, following DA exposure, hippocampal neurons were incubated with Fura-2/acetoxymethyl ester (2 µM) in loading media (Neurobasal Media) for 40 min (37 °C, 5% CO_2_), followed by a 30 min incubation in Krebs-Ringer buffer (0.3 mM CaCl; 131 mM NaCl; 1.3 mM MgSO_4_; 5.0 mM KCl; 0.4 mM KH_2_PO; 6.0 mM glucose; 20 mM HEPES; pH 7.4). Real-time analyses of calcium flux were conducted in 8-well chamber slides mounted on the stage of a Nikon Eclipse TE2000 inverted fluorescence microscope coupled to a digital CCD camera (Hamamatsu Photonics, Bridgewater, NJ, USA) and illuminated with a fluorescent light source. Simultaneous images of cells at *λ*ex 340/380 nm and *λ*em 510 nm were captured at 5 s intervals using Elements software (Nikon) to control a MAC 2000 filter/shutter controller (Ludl Electronic Products, Hawthorne, NY, USA). After approximately 45 s, cells were depolarized by adding 30 mM KCl and image capture continued for 10 min total. Pixel-by-pixel comparisons of the captured images were conducted to generate a ratio of Ca^2+^-bound Fura (340 nm excitation wavelength) to unbound Fura (380 nm excitation wavelength) for each pair of images. Intracellular calcium ([Ca^2+^]_i_) was determined using the method of Grynkiewicz and colleagues [23].

#### Recovery

Cellular recovery (i.e., percent recovery and time to recover) in the hippocampal neurons was evaluated based on calcium clearance ability after depolarization with KCl as described previously [16]. Baseline [Ca^2+^]_i_ levels were determined by averaging [Ca^2+^]_i_ prior to depolarization with KCl. The response to depolarization was defined by the percentage increase of ([Ca^2+^]_i_) over baseline. For all analyses, only cells that demonstrated *>* 40% increase over baseline were considered. Percent increase values, the highest [Ca^2+^]_i_ following depolarization, were determined using the following formula: [(peak − baseline)/(peak) × 100]. Percent cell recovery was determined by assessing the number of cells (within 10 min) in which the [Ca^2+^]_i_ levels returned to 70% of the baseline following KCl depolarization (note: this was either a yes or no response, and therefore a dichotomous variable). Recovery time was determined by assessing how long individual cells took to return to 70% of the baseline. Recovery time was evaluated only for cells that recovered. In this experiment, baseline and responses under the various conditions did not differ, so only the percent recovery from baseline was reported.

### 4.4. HAPI Cell Culture

Highly aggressive proliferating immortalized (HAPI) rat microglial cells were generously provided by Dr. Grace Sun (University of Missouri, Columbia, MO, USA). The HAPI cells were maintained in DMEM supplemented with 10% fetal bovine serum (FBS), 100 U/mL penicillin, and 100 μg/mL streptomycin at 37 °C in a humidified incubator under 5% CO_2_. For the experiments, cells were split into 100 mm plates at a seeding density of 2.5 × 10^6^ cells/well and pre-treated with DMEM containing the EBC combination, EGCG, CURC, BSP, or no supplement for 5 days prior to exposure to a bacterial endotoxin and inflammatory stressor, lipopolysaccharide (LPS, Sigma-Aldrich, St. Louis, MO, USA). On day 3, the media was removed and replaced with fresh media containing the respective treatment. On day 5, the pre-treatment media was removed, and cells were split into 12-well plates and stimulated for 18 h overnight with LPS at 0 or 100 ng/mL in serum-free DMEM without phenol red. For each of the four experiments, treatments were performed in duplicate.

### 4.5. Nitrite Quantification

Following the LPS stimulation of the HAPI cells, the supernatant from each well was removed and stored at −20 °C until use. To assess the production of nitric oxide (NO) from HAPI cells, the extracellular release of nitrite (NO_2_^−^) was measured by Greiss reagent (Invitrogen, Carlsbad, CA, USA) according to the manufacturer’s instructions. From each supernatant sample, 100 μL was used in duplicate for the assay. Absorbance was read at 548 nm, and the concentration of nitrite was calculated with the linear equation derived from the standard curve generated by known concentrations of nitrite.

### 4.6. TNF-α ELISA

The quantification of tumor necrosis factor-alpha (TNF-α) in HAPI cell-conditioned media was performed with enzyme-linked immunosorbent assay (ELISA, Invitrogen, Carlsbad, CA, USA) according to the manufacturer’s instructions. The TNF-α concentration for each sample was calculated from the linear equation derived from the standard curve of known concentrations of the cytokine.

### 4.7. Western Blots

Following treatments and the removal of the supernatant, the HAPI cells were washed with ice-cold PBS and lysed by agitation in CelLytic-M Cell Lysis Reagent (Sigma-Aldrich, St. Louis, MO, USA). Cells were then centrifuged at 18,000× *g* for 5 min to remove any remaining cell debris while the supernatant lysate was used for making samples to be used in blotting. Protein concentrations of the lysates were quantified using the DC protein assay kit (Bio-Rad; Hercules, CA, USA). Equal amounts of denatured protein samples were used for gel electrophoresis. Western blots were performed as described by Poulose and colleagues [24], except that 10% polyacrylamide gels were used. Primary antibodies for iNOS (Millipore, Billerica, MA, USA) and COX-2 (Santa Cruz, Dallas, TX, USA) were used at 1:1000 dilution for incubation overnight at 4 °C. Following primary antibody incubation, the blots were washed with TBST (Tris Buffered Saline/0.5% Tween-20) 3 × 10 min, and then incubated with the appropriate HRP-conjugated secondary antibody (Millipore, Billerica, MA, USA) diluted in RapidBlock. The signal was detected using an electrochemiluminescence (ECL) detection kit (BioRad, Hercules, CA, USA), and the optical density of antibody-specific bands was analyzed by the VisionWorks LS image acquisition and analysis software (UVP, Upland, CA, USA).

### 4.8. Data Analysis

All statistical analyses were performed using SYSTAT software (SPSS, Chicago, IL, USA). Data are expressed as mean (% calcium recovery) or mean ± standard error of the mean (SEM). The data were analyzed by ANOVA followed by post-hoc testing with Fisher’s LSD test to determine differences among groups, except for percent recovery which was analyzed by the Kruskal–Wallis one-way analyses of variance (ANOVA) and Mann–Whitney U post-hoc tests. Results were considered statistically significant if the observed significance level was *p* < 0.05. For each inflammation marker in microglia, cells treated with LPS alone showed statistically higher values for each dependent measure than the control conditions without LPS (unpublished observations). Additionally, pre-treatment with the phytochemical compounds did not significantly affect cells in the absence of LPS in any of the endpoints assayed (unpublished observations).

## Data Availability

Data available upon request.
